# Role of P2X7 and P2Y_2_ receptors on α-secretase-dependent APP processing: Control of amyloid plaques formation “*in vivo*” by P2X7 receptor

**DOI:** 10.1016/j.csbj.2015.02.005

**Published:** 2015-03-11

**Authors:** M. Teresa Miras-Portugal, Juan I. Diaz-Hernandez, Rosa Gomez-Villafuertes, Miguel Diaz-Hernandez, Antonio R. Artalejo, Javier Gualix

**Affiliations:** Biochemistry Department, School of Veterinary Sciences, Complutense University of Madrid, Madrid, Spain, Institute of Neurochemistry (IUIN), Complutense University of Madrid, Madrid, Spain; Health Research Institute of the Hospital Clínico San Carlos (IdISSC), Madrid, Spain

**Keywords:** Alzheimer's disease, APP processing, α-Secretase, GSK-3, P2X7 receptor, P2Y_2_ receptor

## Abstract

Amyloid precursor protein (APP) is expressed in a large variety of neural and non-neural cells. The balance between non-pathogenic and pathologic forms of APP processing, mediated by α-secretase and β-secretase respectively, remains a crucial step to understand β-amyloid, Aβ42 peptide, formation and aggregation that are at the origin of the senile plaques in the brain, a characteristic hallmark of Alzheimer's disease (AD). In Neuro-2a, a neuroblastoma cell line that constitutively expresses APP, activation of the P2X7 receptor leads to reduction of α-secretase activity, the opposite effect being obtained by P2Y_2_ receptor activation.

The *in vivo* approach was made possible by the use of J20 mice, a transgenic mouse model of familial Alzheimer's disease (FAD) expressing human APP mutant protein. This animal exhibits prominent amyloid plaques by six months of age. *In vivo* inhibition of the P2X7 receptor induced a significant decrease in the number and size of hippocampal amyloid plaques. This reduction is mediated by an increase in the proteolytic processing of APP through α-secretase activity, which correlates with an increase in the phosphorylated form of GSK-3, a less active form of this enzyme. The *in vivo* findings corroborate the therapeutic potential of P2X7 antagonists in the treatment of FAD.

## 1 Introduction

Alzheimer's disease (AD) is a progressive and irreversible neurodegenerative disorder characterized by learning and memory impairments, being the most common form of senile dementia. The main neuropathological characteristics of this disease are the accumulation of intracellular neurofibrillary tangles and extracellular amyloid deposits also known as senile plaques in brain [1]. The amyloid precursor protein (APP) is a single helix transmembrane protein expressed in neural and non-neural tissues, being processed in two different ways by sequential proteases, known as secretases [2,3]. In brain, sequential proteolysis of APP by β- and γ-secretases is at the origin of β-amyloid peptide, Aβ42 peptide, which is the most prominent component of extracellular amyloid deposits. The APP protein can also be processed in a non-amyloidogenic way mediated by α- and γ-secretases. Both types of APP processing occur in the central nervous system (CNS), even in the same cell [4], and many questions arise concerning what are the physiological signals that keep a necessary balance between both APP processing ways to avoid an increase of the amyloidogenic pathway in normal brain [5–7].

Neurofibrillary tangles are the intracellular companion of senile plaques in AD. These inclusions contain the microtubule-associated protein, tau, in its hyperphosphorylated forms. This protein, which is mostly found in neurons, stabilizes microtubules and can be phosphorylated by multiple kinases, among them glycogen synthase kinase 3, GSK-3. When the balance between phosphorylation and dephosphorylation is inaccurate, tau self-assembly occurs. Although most of the effects reported for tau are at the intracellular level [8], it has been recently reported that extracellular tau and its peptide fragments behave as non-desensitizing agonists on muscarinic M1 and M3 receptors, with a sustained cytosolic Ca^2 +^ increase in neural cells [9,10]. On the other hand, phosphorylated tau or its fragments have to be dephosphorylated to be fully active on muscarinic receptors. It is relevant that one of the ecto-phosphatases involved is the tissue non-specific alkaline phosphatase, TNAP, which is already reported to play a role on axonal growth [9–14]. These findings constitute an example about the complexity of Alzheimer's disease, in which a clear hereditary transmission has only been demonstrated for the FAD early-onset genes, APP and presenilins PS1 and PS2, whereas the ε4 allele of the APOE gene increases the risk of developing the disease. The meta-analyses and genome-wide association studies are now contributing to the approach and understanding of the sporadic, also known as late-onset, AD. These studies allowed the identification of a large number of genes, some related with innate immunity, cellular signaling or Aβ clearance, which has contributed to open the field to new views and interpretations [15,16].

β-amyloid peptide production and aggregation is still considered to be at the origin of Alzheimer's pathology. The possibility of enhancing the non-amyloidogenic APP processing by different G protein-coupled neurotransmitter receptors (GPCRs) has been postulated, and modulation of the α-, β- and γ-secretases action on APP by diverse signaling cascades has been proved [17].

GSK-3, which plays a role in multiple signaling pathways, is among the enzymes that can influence Aβ production. It has been reported that an increase in GSK-3 activity [18] correlates with the phosphorylation of APP intracellular domain, making it a more suitable substrate for γ-secretase proteolysis, which enhances Aβ-42 production [19]. Moreover, as already mentioned, GSK-3 is one of the enzymes responsible for tau phosphorylation [20], thus becoming a link between senile plaques and neurofibrillary tangle formation.

Among neurotransmitters, nucleotides play a relevant role through the activation of ionotropic P2X and metabotropic P2Y receptors. These receptors are widely distributed in CNS, where they regulate calcium homeostasis, neurotransmitter release and a broad diversity of intracellular signalling pathways involved in brain physiology and pathophysiology [21]. The P2X7 receptor, which is abundantly expressed in CNS, being present in diverse cellular subtypes such as microglia, astrocytes or neurons, has emerged as a relevant target among the P2 receptors family [22–25]. Early reports demonstrated the up-regulation of the P2X7 receptor in microglial cells in neuroinflammatory situations, and the beneficial effect of its antagonism [26]. In addition, P2X7 receptor is receiving special attention in neurodegenerative diseases, such as Huntington's and Parkinson's disease [27,28]. The role of P2X7 receptor has been more intensively studied in Alzheimer's disease, where the effects of the signalling cascades coupled to P2X7 activation on APP processing have been reported [29,30]. Other P2 receptors, as it is the case for P2Y_2_ receptor, have also been involved in APP processing regulation, mainly through activation of the non-amyloidogenic pathway [31–33]. In this review the role of P2X7 and P2Y_2_ receptors on the APP processing will be discussed, together with the relevance of experimental models and the possibility of use of those receptors as valuable therapeutic targets.

## 2 Neuro-2a cells as a model for APP processing: Effect of P2Y_2_ and P2X7 receptors activation

**Fig. 1:**
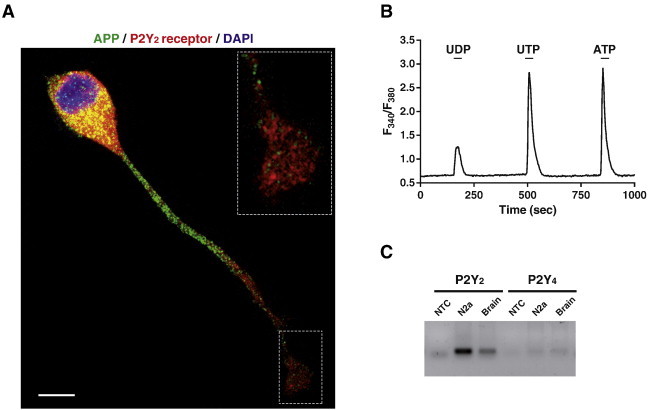
APP and functional P2Y_2_ receptors are co-expressed in N2a cells. (A) N2a cells were cultured in 0.5% fetal bovine serum-containing medium for 4 days in the presence of 1 mM DiBucAMP. Afterwards, cells were fixed and immunostained with anti-amyloid β clone WO2 (green) antibody from Millipore and anti-P2Y_2_ receptor (red) antibody provided by Alomone Labs. Nuclei were counterstained with DAPI (blue). Scale bar, 10 μm. Insert shows magnification of neurite distal region ending in a growth cone-like structure, where high levels of P2Y_2_ receptor can be observed. (B) Intracellular calcium increments evoked by 30 sec stimulation with 100 μM UDP, UTP, or ATP in N2a cells. Horizontal bars indicate stimulation periods. Traces represent mean from 150 individual cells. (C) Expression of P2Y_2_ and P2Y_4_ receptors was analyzed by RT-PCR in both N2a cells, and adult whole mouse brain mRNA extracts. NTC, non-template control. For methods see Ref. [30]. (For interpretation of the references to colour in this figure legend, the reader is referred to the web version of this article.)

The Neuro-2a cell line, N2a, has been largely employed as a neural model to study signalling pathways, secretory events and neuronal differentiation, thus being a well characterized system [34,35]. These cells have the advantage of constitutively expressing APP, together with functional P2Y_2_ and P2X7 receptors. P2Y_2_ receptors are abundant in N2a cells, exhibiting a broad distribution, which can be observed even when neural-like differentiation is induced, as shown in Fig. 1A. The same figure shows the APP distribution along the axon until reaching the axonal growth cone, where a great abundance of the P2Y_2_ receptor can be observed. This distribution agrees with a recent fluorescence-based procedure allowing the study of the axonal transport of APP in cultured hippocampal neurons [36]. The absence of P2Y_4_ receptor in N2a cells (Fig. 1C), which exhibits a similar agonistic profile as the P2Y_2_ receptor (Fig. 1B), clearly substantiates a role for P2Y_2_ receptor activation in APP processing.

**Fig. 2:**
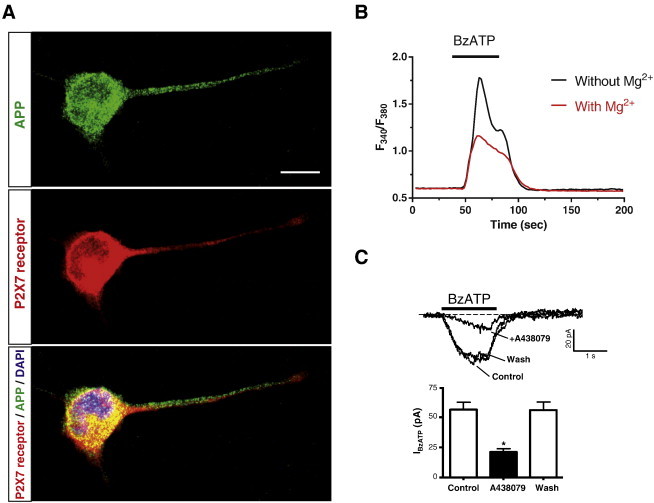
APP and functional P2X7 receptors are co-expressed in N2a cells. (A) N2a cells were cultured in 0.5% fetal bovine serum-containing medium for 4 days in the presence of 1 mM DiBucAMP. Afterwards, cells were fixed and immunostained with anti-amyloid β clone WO2 (green, upper panel) antibody and anti-P2X7 receptor (red middle panel) antibody from Alomone Labs. Merge image with DAPI-labelled nucleus (blue) is shown (lower panel). Scale bar, 10 μm. (B) Intracellular calcium increments elicited by 100 μM BzATP in N2a cells are potentiated in the absence of extracellular Mg^2+^ ions. Horizontal bar indicates stimulation period. Trace represents mean from 100 individual cells. (C) Effect of 1 μM A438079 on whole-cell current responses evoked by 100 μM BzATP in N2a cells. Top panel: current responses to BzATP in the absence (Control; Wash) and presence of A438079 (+ A438079). BzATP was applied at 5 min intervals and A438079 was superfused 2 min before and during the second BzATP application. Bottom panel: peak current amplitudes evoked by successive applications of BzATP in the absence or presence of A438079 (four cells). Drugs were administered during the time indicated by horizontal bars. Broken lines denote the zero current level. V_h_ = − 70 mV. *p < 0.05, unpaired Student's *t* test. For methods see Ref. [34]. (For interpretation of the references to colour in this figure legend, the reader is referred to the web version of this article.)

P2X7 receptor immunolabelling shows a distribution similar to that observed for APP, being present not only in the neuronal cell body, but also in the axon-like extension [37,38] (Fig. 2A). The presence of a functional P2X7 receptor has been demonstrated by calcium imaging fluorescence techniques, challenging neural cells with the selective P2X7 receptor agonist benzoyl ATP (BzATP) in the presence or absence of Mg^2+^ ions (Fig. 2B), and also by electrophysiological techniques in which the current elicited by stimulation with BzATP and the inhibition exerted by the specific reversible antagonist A438079 were measured (Fig. 2C).

APP processing in N2a cells can be followed by detection of the specific proteolytic fragments present in the plasma membranes. The presence of the carboxy-terminal C83 fragment, α-CTF, indicates that membrane protein APP has been processed by α-secretase, which results in the simultaneous release of the extracellular protein moiety, APPsα. The C83 fragment is further hydrolysed by γ-secretase that cleaves the carboxy-terminal fragment in the middle of APP transmembrane helix, which results in the release of the extracellular peptide, P3, and an intracellular C-terminal fragment, AICD. The AICD fragment is identical in both the amyloidogenic and non-amyloidogenic APP processing, and a role on the control of gene expression has been postulated for this fragment [39].

**Fig. 3:**
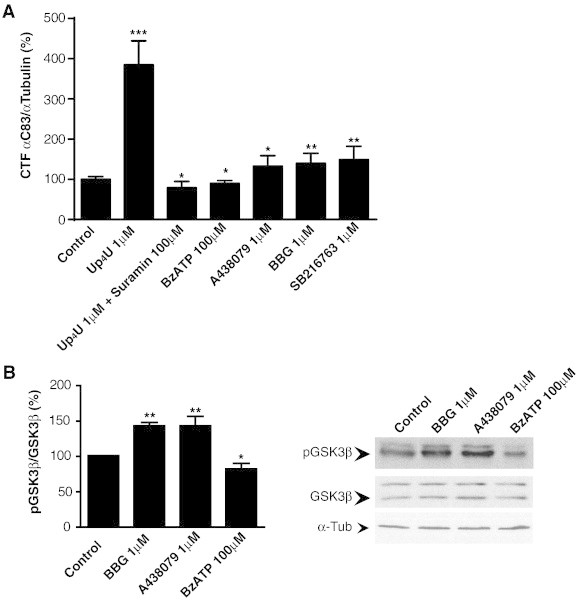
Purinergic receptors regulate α-secretase and GSK-3 activities in N2a cells. (A) Protein levels of CTF C83 detected in N2a cells treated with the P2Y_2_R agonist Up_4_U (1 μM), both suramin (100 μM) and Up_4_U (1 μM), BzATP (100 μM), A438079 (1 μM), BBG (1 μM), or SB216763 (1 μM). Histogram represents the mean ± SEM of CTF C83/α-tubulin ratios normalized to control untreated cells (n = 4 independent experiments in duplicate). (B) Western blot detection of p-GSK-3β (pSer9) and total GSK-3 in N2a cells treated with BBG (1 μM), A438079 (1 μM) or BzATP (100 μM). Histogram represents the mean ± SEM of p-GSK-3β/total GSK-3β ratios (n = 3 independent experiments in duplicate). In all cases, α-tubulin was used as loading control, and ratios were normalized to control untreated cells (100%). *p < 0.05, **p < 0.01, ***p < 0.005 compared to control using ANOVA with Dunnet´s post-test analysis. For methods see Ref. [49].

P2Y_2_ receptor agonists are able to significantly increase the α-secretase-mediated APP processing in N2a cells, this stimulatory effect being consequently blocked by the broad spectrum P2 antagonist, suramin, as shown in Fig. 3A. These results agree with those obtained by other groups (as Gary Weisman's group), supporting the role of P2Y_2_ receptor in neuroprotection, an effect that is mediated at least in part via the activation of the APP non-amyloidogenic pathway through α-secretase processing [31–33,40].

The activation of P2X7 receptor in N2a cells decreases the levels of C83 fragment, which is the indicator of the α-secretase non-amyloidogenic hydrolytic pathway of APP processing (Fig. 3A). This effect can be overturned by using P2X7 receptor antagonists; both the reversible and more specific A438079 and the wider spectrum less specific Brilliant blue G (BBG) were able to increase the C83 fragment product of α-secretase (Fig. 3A). These results contrast with those obtained by other authors, but it is relevant to emphasize that APP processing depends on the abundance of this protein at the specific cellular model and in addition, when it is overexpressed, the equilibrium between the different proteolytic pathways could be unbalanced, making it more difficult to understand the process [29,41].

The effect of P2X7 receptor-activated signaling cascades on GSK-3 activity has been already reported by several authors. Activation of P2X7 receptor in granule cells from cerebellum results in GSK-3 phosphorylation through the PI3K/AKT cascade, which in turn promotes neuroprotection [23,42]. However, P2X7 receptor activation induces a reduction of GSK-3 phosphorylation and stops axonal elongation in embryonic hippocampal neurons during differentiation, both effects being reverted by P2X7 receptor antagonists or by the reduction of the extracellular levels of ATP ligand by the use of alkaline phosphatase [38]. N2a cells, behave in a similar way to cultured hippocampal neurons, as stimulation with P2X7 receptor agonist results in a decrease of GSK-3 phosphorylation in serine 9/21 as shown in Fig. 3B, which correlates with a reduction in the α-secretase-generated C83 APP fragment (Fig. 3A). Consequently, the P2X7 receptor antagonists, A438079 and BBG, significantly increased GSK-3 phosphorylation and production of C83 fragment (Fig. 3A and B). A similar effect was obtained with the GSK-3 inhibitor SB216763 (Fig. 3A), thus corroborating the pharmacological relevance of the inhibitors of this enzyme in AD.

## 3 P2X7 receptor and its role in animal models of Familial Alzheimer's Disease

There are many models of genetically modified mice that develop cerebral amyloid deposits. The transgenic mice known as J20 hAPP have been chosen because they develop the characteristic amyloid peptide deposits by 6–8 months of age. These transgenic mice, labelled as B6.Cg-Tg(PDGFB-APPSwInd)20Lms/2J strain, express a mutant form of the human amyloid protein precursor bearing both the Swedish (K670N/M671L) and the Indiana (V717F) mutations (APPSwInd), [43]. All procedures were carried out in accordance with European and Spanish regulations (86/609/CEE; RD1201/2005) when working with these animals in our laboratory.

The main question was to understand the balance between both APP proteolytic pathways in *in vivo* situations and whether it was possible to change the dynamics of amyloid deposits by impacting P2Y_2_ and P2X7 receptors.

Concerning P2Y_2_ receptor, there are not selective agonists or antagonists with good pharmacokinetics parameters for *in vivo* administration to date. However, its relevance has been confirmed in the TgCRND8 mouse model of Alzheimer's disease, where loss of P2Y_2_ nucleotide receptors enhances the β-amyloid (Aβ) deposit and also the soluble Aβ1–42 levels in the cerebral cortex and hippocampus [31].

The availability of P2X7 receptor ligands for *in vivo* studies is slightly better, as the antagonist BBG is able to infiltrate the brain parenchyma. The efficacy of this antagonist in mice has been already reported in the beneficial effects on Huntington's disease symptomatology and the seizure suppression and neuroprotection in status epilepticus [27,44]. Recently, BBG has proved to improve cognition in an animal model of Alzheimer's disease [45]. In addition to BBG, there are many other P2X7 receptor antagonists able to reach the brain, as it is the case of A438079 [46].

The hippocampi of the J20 mice showed abundant amyloid plaques at the age of 6–8 months. These deposits were clearly identified with anti-Aβ antibodies and the Thioflavin-T dye that is able to intercalate between the β-sheet structures of amyloid deposits. To study the role of P2X7 receptor on β-amyloid deposits *in vivo*, J20 mice were treated before the appearance of the first hippocampal plaques, at the age of 4 months, with BBG (intraperitoneally injected every 48 hours at 45.5 mg/Kg), or vehicle solution, PBS, for 4 months. BBG concentration attained in brain was around 200 nM at the dose used. It is relevant to emphasize that the concentration reached *in vivo* is in the range of the IC_50_ of BBG to antagonize P2X7 receptor (10–200 nM) [47].

**Fig. 4:**
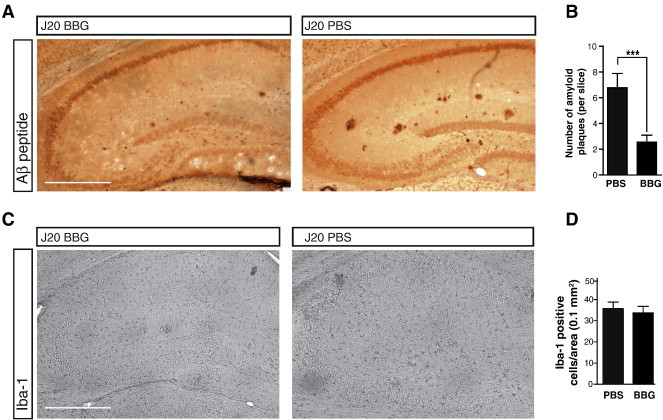
Brilliant Blue-G (BBG) treatment reduces the number of amyloid plaques and microglia in the J20 mouse hippocampus. (A) Immunostaining of WO2 in hippocampal slices from 6 to 8 months-old J20 mice injected intraperitoneal with vehicle or BBG. Scale bar, 500 μm. (B) Histogram represents the mean ± SEM of Aβ-amyloid plaques per slice in the hippocampus of J20 mice treated with vehicle or BBG, being 16 slices per mouse (n = 7 mice per condition). ***p < 0.005, unpaired Student's *t* test. (C) Immunostaining of microglial marker Iba-1 in hippocampal slices from 6 to 8 months-old J20 mice treated with vehicle or BBG. Scale bar, 500 μm. (D) Quantification of microglial cells in the hippocampus of J20 mice treated with vehicle or BBG. Histograms represent the mean ± SEM of microglial cells per hippocampal area of 0.1 mm^2^ being 16 slices per mouse (n = 7 mice per treatment). For methods see Ref. [49].

After BBG treatment the number and size of amyloid plaques at the hippocampal structures of J20 mice were significantly reduced compared to their littermates treated with vehicle, as shown in Fig. 4A and B. In addition, the treatment with BBG did not significantly modify either the P2X7 receptor or murine APP and human APP mRNA expression. The levels of these proteins and their distribution patterns in the hippocampus were also not modified by the treatment with BBG, as demonstrated by western blot and immunohistochemical studies. However, a dramatic change was observed concerning the pattern of the C83 and C99 peptides, which correspond to the carboxyterminal fragments generated by APP cleavage by α-secretase and β-secretase, respectively. C99 fragment was under the limits of detection in wild type mouse brain, but was very abundant in the brain of J20 mice, which exhibit a much lower concentration of the α-secretase generated C83 fragment. However, BBG reversed the situation and a significant increase in the C83 fragment was achieved in the brain of BBG-treated J20 mice [48]. This situation mimics the APP processing pattern observed in N2a cells treated with P2X7 receptor inhibitors. On the other hand, an increase in the phosphorylated form of GSK-3 was observed in the hippocampus of BBG-treated J20 mice, when compared with vehicle-treated animals. Thus, BBG was able to reduce hippocampal GSK-3 activity in FAD animal models in the same way as in N2a cells.

J20 animals at the end of their lives, about 20 months-old, exhibit a profusion of hippocampal senile plaques that were surrounded by microglial cells. However, at the first stages of the FAD neurodegenerative disease a significant presence of microglial positive cells cannot be observed when considering the total hippocampal structures (Fig. 4C and D). Only some few cells surrounding the senile plaques expressed microglial markers, together with P2X7 receptor and hypophosphorylated GSK-3. This fact is relevant because to date, most of the effects of P2X7, and also P2Y_2_, receptors have been explained by microglia activation around the senile plaques due to the amyloid Aβ-42 peptide or the release of extracellular nucleotides and cytokines, thus helping in the extracellular clearance of the amyloid deposits [26,29,31,49].

## 4 Summary and Outlook

In spite of the complexity of Alzheimer's disease physiopathology, it is however relevant to tackle one of the main characteristics of the disease, the formation of amyloid plaques, using an *in vivo* mouse model of FAD. In this model, it was demonstrated for the first time that the *in vivo* inhibition of P2X7 receptors significantly reduces the amyloid plaques formation in brain hippocampal structures. The molecular mechanisms underneath this relevant effect, reported here, were the phosphorylation and consequent reduction of GSK-3 activity, which correlates with an increase in α-secretase activity. Apparently, the prolonged BBG treatment is efficient and non-toxic, thus providing a suitable therapeutic approach to prevent amyloid deposition on FAD. However, remarkable differences exist when studying the effects of P2X7 receptor agonists and antagonists in various neural cell models. N2a cells and primary cultures of embryonic hippocampal neurons, behave as the adult hippocampus neurons regarding APP processing. By contrast, P2X7 receptor activation in cultured cerebellar granule neurons results in GSK-3 inhibition and neuroprotection. Consequently, although significant progress has been made over the past decade understanding AD and β-amyloid deposit, it is relevant to emphasize that the brain is a complex entity where many different structures coexist.
